# Targeting EIF3C to suppress the development and progression of nasopharyngeal carcinoma

**DOI:** 10.3389/fbioe.2022.994628

**Published:** 2022-09-06

**Authors:** Qian Zhao, Xuehui Luo, Honghui Li, Yanxia Bai, Qian Chen, Ming Yang, Bei Pei, Chongwen Xu, Suxia Han

**Affiliations:** ^1^ Department of Otorhinolaryngology Head and Neck Surgery, The First Affiliated Hospital of Xi’an Jiaotong University, Xi’an, Shaanxi, China; ^2^ Department of Radiation Oncology, The First Affiliated Hospital of Xi’an Jiaotong University, Xi’an, Shaanxi, China

**Keywords:** EIF3C, cancer therapy, head and neck cancer, pharyngeal cancer, nucleic acid drug

## Abstract

Nasopharyngeal carcinoma occurs in many parts of the pars nasalis pharyngis, and the pathological type is mainly squamous cell carcinoma. Because of the special position of nasopharynx, breathing, pronunciation and daily life will be seriously affected. At present, the research direction of nasopharyngeal carcinoma is mainly to explore the law of tumor cell proliferation and migration, study the molecular mechanism, master its biological behavior and clinical significance, try to find therapeutic targets, and further improve the level of tumor treatment. However, the pathologic structure and molecular mechanism of nasopharyngeal carcinoma have not been fully elucidated. In this study, the Lentivirus-mediated EIF3C shRNA vector (L.V-shEIF3C) was constructed to down-regulate the expression of EIF3C in human pharyngeal squamous carcinoma cell FaDu and the human nasopharyngeal carcinoma cell 5-8F, it was found that down-regulation of EIF3C could significantly inhibit the cell proliferation, promote cell apoptosis, induce cell cycle arrest, and inhibit the formation and growth of tumors in mouse models. This study provides strong evidence that EIF3C is a key gene driving the development and progression of head and neck cancer, which is of great significance for the diagnosis, prognosis or treatment of tumors, suggesting that EIF3C may become a valuable therapeutic development and intervention target.

## Introduction

Head and neck cancer is the sixth most common malignant tumor in the world (Hsieh et al., 2019). According to statistics, there were about 700,000 cases and 350,000 deaths of lip, oral, oropharyngeal, and hypopharyngeal cancers worldwide in 2020 (Sung et al., 2021). Nasopharyngeal carcinoma (NPC) and head and neck squamous cell carcinoma (HNSCC) were broadly defined as head and neck cancer (HNC) (Luo et al., 2018). The pathological types of head and neck tumors were mainly squamous cell carcinoma, which can occur in many parts of head and neck, including larynx, thyroid, nasopharynx, and maxillofacial (Johnson et al., 2020). Although head and neck tumors account for only about 4% of malignancies in all parts of the body, a wide range of symptoms can occur in this relatively small area (Aupérin, 2020). The head and neck organs were inextricably linked to vital basic physiological functions, including appearance, expression, breathing, nutrition and social interaction (Kristensen et al., 2020). Different sites, tumor size, invasion mode and treatment complications of head and neck tumors may lead to different degrees of structural damage and dysfunction, which can significantly reduce the quality of life of patients (Gavrielatou et al., 2020).

Oral squamous cell carcinoma is the most common and malignant oral-maxillofacial tumor, which etiology has certain relevance with bad habits, and may be associated with excess of alcohol, tobacco, and nutritional factors (Kawakita and Matsuo, 2017). Furthermore, the cancer cells generally have low differentiation, strong invasion and metastasis ability making early detection difficult, which results in the middle and late stage at the time of diagnosis (Saloura et al., 2013). In addition, there are many tissues around the larynx and throat, and lymphatic tissue is unusually rich, leading to lymph node tissue metastasis in the early stage of malignant tumor. Studies have shown that about 50% patients had the surrounding lymph node metastasis, the 5 years survival rate less than 50% (Horton et al., 2019).

Nasopharyngeal carcinoma (NPC) is a malignant tumor originating from the mucosal epithelium of the nasopharynx, and is also a common head and neck tumor (Lam and Chan, 2018). According to the latest classification of WHO, NPC is divided into two histological subtypes: non-keratinocarcinoma (differentiated or undifferentiated) and keratinized squamous cell carcinoma (Chen et al., 2019). Compared with common squamous cell carcinoma of the head and neck, nasopharyngeal carcinoma has characteristic clinical manifestations, which is sensitive to radiotherapy and chemotherapy, and has a better prognosis. With the emphasis on the development of radiotherapy technology and the optimization of chemotherapy regimen, the local regional control rate of Nasopharyngeal carcinoma has been significantly improved, but there are still 3%–27% patients with treatment failure (Liao et al., 2020; Rosenberg, 2020; Huang et al., 2021).

Protein synthesis is a complex, multi-step, precisely regulated process of gene translation, which is divided into three stages: initiation, extension and termination. The initiation stage of translation is the most complex stage in the whole translation process, and its core is the initiation control of translation (Jackson et al., 2010). Eukaryotic translation initiation factors (EIFs) are a series of proteins involved in the initiation of eukaryotic protein translation. EIFs interacts with other EIFs, messenger RNA, transport RNA and ribosomes to form a complex interaction network system to participate the initiation of eukaryotic translation. Currently, there are 12 known EIFs, among which eukaryotic translation initiation factor 3 (EIF3), with a molecular weight of about 650 kDa, is the largest and most complex eukaryotic translation initiation factor, specifically involved in the targeted regulation of cell cycle, differentiation, apoptosis, and other biological processes (Lee et al., 2015; Robichaud et al., 2019). Meanwhile, more and more studies have found that EIFs is closely related to the occurrence and development of human diseases, especially tumors. Among them, the core subunit C of eukaryotic translation initiation factor 3 (EIF3C) has been found to be significantly overexpressed in a variety of tumors, including osteosarcoma, cervical cancer, liver cancer and breast cancer (Li et al., 2017; Zhao et al., 2017; Gao et al., 2019; Hu et al., 2019). However, the specific mechanism of EIF3C in head and neck cancer is still unclear.

In order to clarify the biological function of EIF3C in head and neck cancer, this study constructed EIF3C-shRNA lentvirus and inhibited the expression of EIF3C to study the biological effects of EIF3C on the proliferation, growth and apoptosis of FaDu and 5-8F cells. This study provides a theoretical basis for further understanding the molecular mechanism of EIF3C in the occurrence and development of head and neck cancer, and also provides a potential new target for the treatment of head and neck cancer.

## Materials and methods

### General remarks

Minimum essential medium (MEM), RPMI-1640 medium, phosphate buffer saline (PBS), fetal bovine serum (FBS), penicillin streptomycin (Pen Strep), and TRIzol™Reagent were purchased from Fisher Scientific Ltd. Other chemicals used in this study were purchased from Sigma-Aldrich unless otherwise specified.

### Cell culture

The human pharyngeal squamous carcinoma cell line FaDu and the human nasopharyngeal carcinoma cell line 5-8F were purchased from the national Biomedical Cell Resource (BMCR, China). Fadu cells were cultured in MEM medium supplemented with 10% FBS and 1% Pen Strep, 5-8F cells were cultered in RPMI-1640 medium supplemented with 10% FBS and 1% Pen Strep, the two cells were incubated at 37°C in 5% CO_2_.

### Lentiviral-mediated shRNA vector and infection

The lentiviral-mediated EIF3C shRNA vector (L.v-shEIF3C), the negative control shRNA vector (L.v-shCtrl) and transfection reagent Polybrene were purchased from GenePharma (Shanghai, China). 1 × 10^6^ FaDu or 5–8F cells were plated into 6-well plates and were infected with L.v-shEIF3C (MOI = 50) or L.v-shCtrl (MOI = 50) respectively when the cell density reached 30%–40%, 12 h after infection, the medium was replaced with conventional complete medium, 72 h after infection, the cells can be used to the subsequent experiments when transduction efficiency reached 70% or more. The EIF3C and negative control shRNA sequence was as follows:

L.v-shEIF3C: 5′-CCA​TCC​GTA​ATG​CCA​TGA​A-3′.
L.v-shCtrl: 5′-TTC​TCC​GAA​CGT​GTC​ACG​T-3′.

### RNA extraction and quantitative real-time PCR

The total RNA of cells was isolated by Trizol method, and the concentration and purity of which were detected by ultraviolet spectrophotometer. Then, RNA was converted into complementary DNA (cDNA) first with RevertAid First Strand cDNA Synthesis Kit and DNase I (Thermo Scientific™). QRT-PCR was conducted using TB Green qPCR Master Mix (Takara) with LightCycler^®^ 2.0 Real-time PCR System.

The primer sequences of EIF3C were as follows: 5′-AGA​TGA​GGA​TGA​GGA​TGA​GGA​C-3′ (forward) and 5′- GGA​ATC​GGA​AGA​TGT​GGA​ACC-3′ (reverse); the primer sequences of GAPDH were as follows: 5′-TGA​CTT​CAA​CAG​CGA​CAC​CCA-3′ (forward) and 5′-CAC​CCT​GTT​GCT​GTA​GCC​AAA-3′ (reverse).

### Western blot

The total protein of cells was extracted by radio-immune precipitation assay (RIPA) lysis buffer adding protease inhibitors PMSF (Beyotime, China) at a ratio of 100:1 (v/v), the protein concentration was detected by BCA reagent kit (Beyotime). To denature protein, mixing the protein sample with 5× loading buffer at ratio of 4:1 (v/v), boiling the mixed solution at 100°C for 10 min. Then, 30 μg of total protein was loaded into the wells of SDS-PAGE gel to separate, after running time, the gel was transferred to polyvinylidene difluoride (PVDF) membrane and the proteins were detected by different antibodies including antibodies against EIF3C (Abcam, United States) and GAPDH (Proteintech, China). An anti-mouse horseradish peroxidase antibody was used as a secondary antibody. Finally, the PVDF membranes were imaged with the enhanced chemiluminescence reagent (ECL, Millipore) by a chemiluminometer (BioRad, United States).

### Cell growth assay

2 × 10^3^ FaDu and 5-8F cells infected with L.v-shEIF3C or L.v-shCtrl were planted in 96-well plate and incubated overnight, each group had three replicates. The plate was read and the cells were photographed by Cellomics (Thermo) for five consecutive days, to accurately calculate the number of cells with green fluorescence in hole plates by adjusting the input parameters of Cellomics ArrayScan, the data were statistically plotted and the 5-days cell proliferation curve was drawn.

### Colony formation assay

The FaDu and 5-8F cells infected with L.v-shEIF3C or L.v-shCtrl were counted, and each group 500 cells were plated in six-well plates with three replicates. After 2 weeks of culture, the cell colonies were fixed with methanol for 20 min and were subsequently stained with a 0.5% crystal violet staining solution for 20 min. Finally, the cell colonies were photographed and counted.

### BrdU cell proliferation assay

4 × 10^4^ FaDu cells infected with L.v-shEIF3C or L.v-shCtrl were seeded at varying density in serum free medium in a 96-well plate and incubated overnight, then the medium was replaced with complete medium adding 10% serum and the cells were incubated for 24 h. Next, 10 μl bromodeoxyuridine (BrdU, Merck) was added to the plate and cells were incubated for 4 h, after removing labeling medium, cells were fixed by FixDenat 200 μl/well for 30 min, a BrdU mouse mAb was then added to detect the incorporated BrdU, and substrate solution was added to develop color. Finally, the magnitude of the absorbance for the developed color can be detected at 450 nm with a microplate reader (Tecan infinite).

### Cell cycle analysis with flow cytometry

2 × 10^5^ FaDu and 5-8F cells infected with L.v-shEIF3C or L.v-shCtrl were planted in 6-well plate and incubated for 48 h, the cells were collected and fixed with 70% ice-cold ethanol overnight, then stained with 0.3 ml PI/RNase Staining Buffer (BD Biosciences) for 15 min and analyzed by flow cytometry.

### Apoptosis analysis with flow cytometry

FaDu and 5-8F cells infected with L.v-shEIF3C or L.v-shCtrl were collected and washed twice with cold PBS and then resuspended in 1X Binding Buffer at a concentration of ∼1 × 10 ^6^ cells/ml, then stained with 5 μl Annexin V and 5 μl Vital Dye 7-AAD (BD Biosciences) for 15 min in the dark and analyzed by flow cytometry.

### PathScan sandwich ELISA

Using Cell Signaling Technology (CST) PathScan^®^ Antibody Array Kit to detect and compare the changes of key signal molecules in the signal pathway in the samples. FaDu cells infected with L.v-shEIF3C or L.v-shCtrl were collected and lysed by cell lysate plus protease inhibitor, the cell lysate was then added to the prepared test well plate, 70 µl per well at 4 Covernight. After the cell samples were removed on the second day, the plate was washed three times with PBS buffer, and then 1X antibody detection mixture was added for incubating at room temperature, after 1 h, the plate was cleaned again, 1X HRP-linked streptomavidin was subsequently added for incubating for 0.5 h. Finally, chemiluminescence was performed.

### FaDu cell line derived tumor xenograft mouse model

Four-week-old female BALB/c nude mice were purchased from Beijing Vital River Laboratory Animal Technology (Beijing, China). The nude mice were randomly divided into two groups, each group had 10 mice. FaDu cells were transfected with L.v-shEIF3C or L.v-shCtrl according to Multiplicity of Infection (MOI) 50:1 for 4 days. Then, the cells were collected and resuspended in PBS at a concentration of 2 × 10^7^ cells/ml, we subcutaneously injected 200 μl cell suspension into the back of the nude mice. After 14 days, we measured the longest and shortest diameters of tumors every 4 days with calipers, which were, respectively, recorded as L and W, and the tumor volumes were calculated as V = L × W^2^ × 0.5. After 32 days, before the mice were sacrificed, we used a small-animal *in vivo* imaging system to detect the fluorescence of tumors in nude mice, and then the mice were photographed, the tumors were taking out and weighted.

## Results

### L.v-shEIF3C significantly decreased the expression of EIF3C in FaDu and 5-8F cells

**FIGURE 1:**
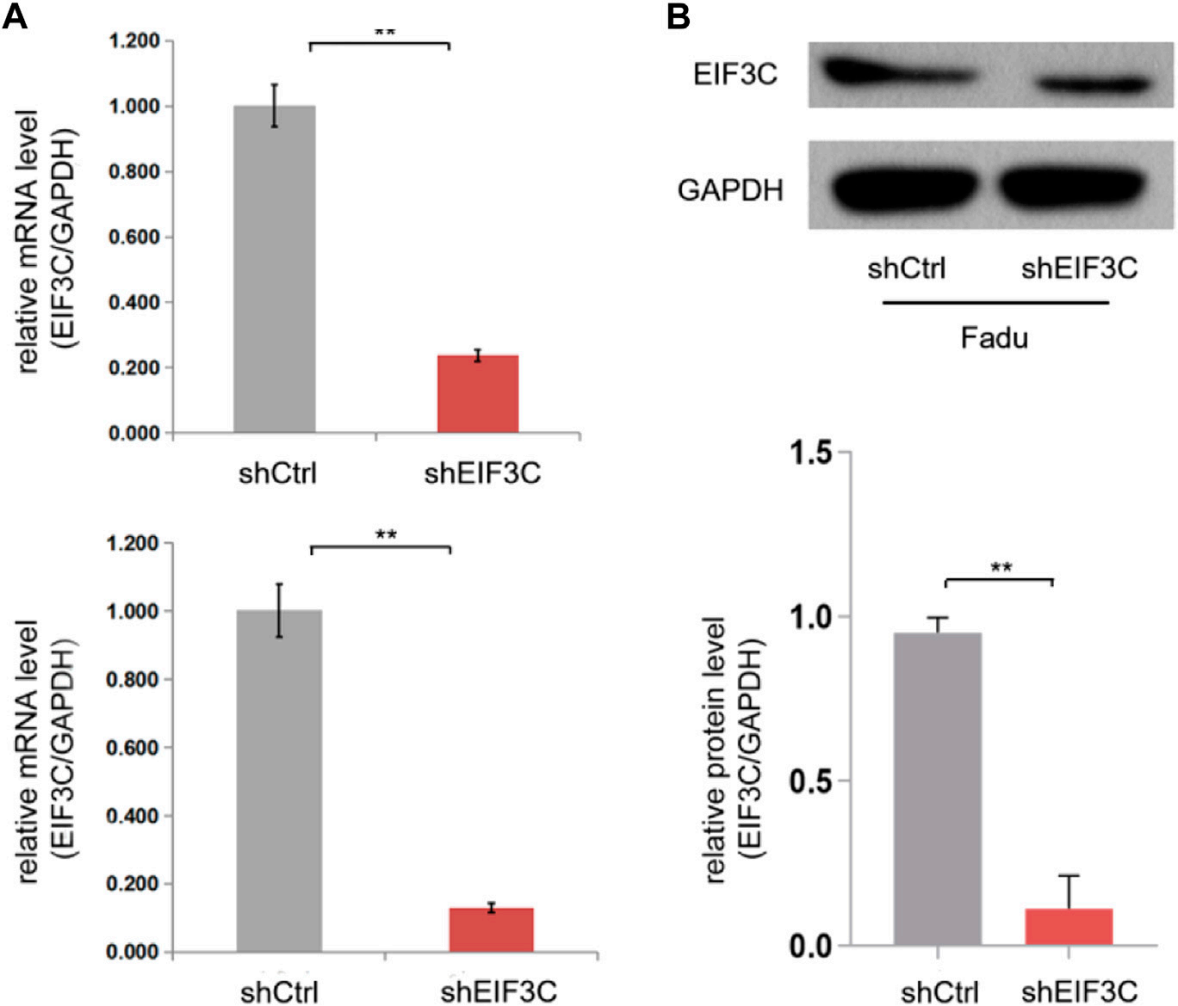
The L.v-shEIF3C decreased the expression of EIF3C in FaDu and 5-8F cells. **(A)** qRT-PCR analysis showed that the L.v-shEIF3C significantly decreased the mRNA expression of EIF3C in FaDu and 5-8F cells. **(B)** Western blot showed that the L.v-shEIF3C significantly decreased the protein expression of EIF3C in FaDu cells, the grey scale of the protein bands was analyzed with ImageJ software. The data is the means ± SDs of three independent experiments. The differences were statistically significant. **p < 0.01.

In order to research the role of EIF3C in head and neck carcinoma, the lentiviral-mediated EIF3C shRNA vector (L.v-shEIF3C) and the negative control shRNA vector (L.v-shCtrl) were constructed to infect the human pharyngeal squamous carcinoma cell line FaDu and the human nasopharyngeal carcinoma cell line 5-8F. The RNA (Figure 1A) and protein (Figure 1B) expression of EIF3C in cells were respectively detected by qRT-PCR and western blotting, the results showed that L.v-shEIF3C could significantly down-regulate the expression of EIF3C in FaDu and 5-8F cells. Next, we used this strategy to study the effects of EIF3C on the proliferation, apoptosis and cell cycle of head and neck carcinoma cells.

### Down-regulation of EIF3C inhibited the proliferation of FaDu and 5-8F cells

**FIGURE 2:**
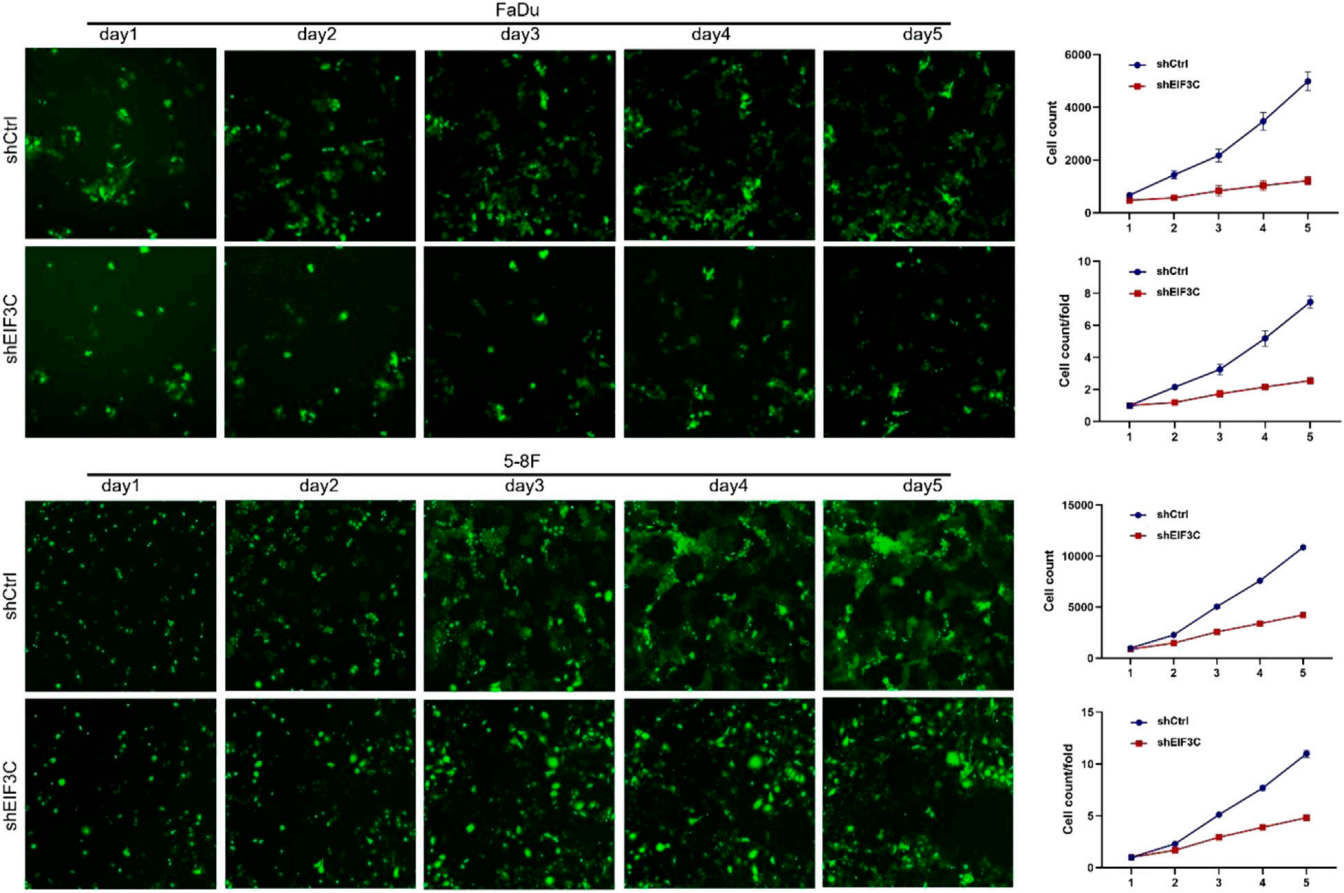
The L.v-shEIF3C inhibited the proliferation of FaDu and 5-8F cells in cell growth assay. For five consecutive days, the images and the statistics exhibited that the number of cells with fluorescence in the L.v-shEIF3C group was less than that in the L.v-shCtrl group. The differences were statistically significant.

**FIGURE 3:**
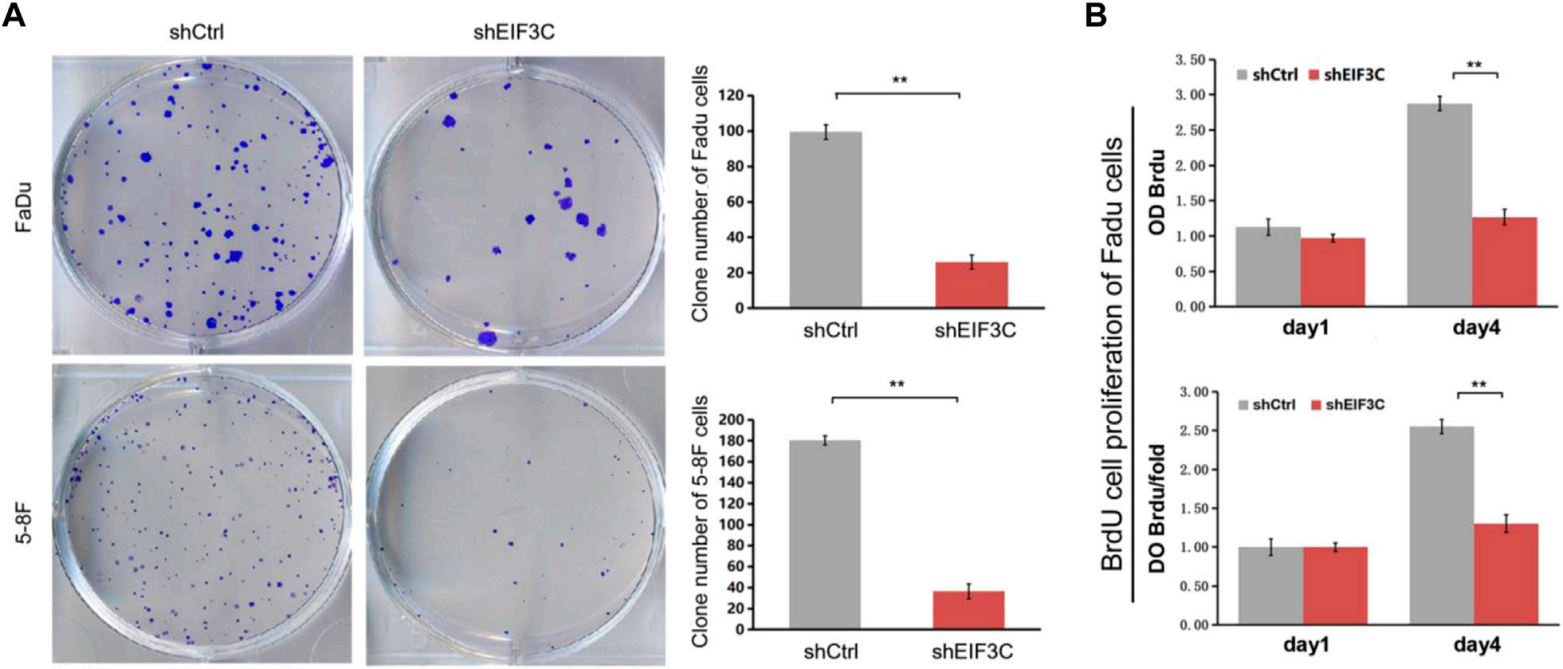
The L.v-shEIF3C inhibited the proliferation of FaDu and 5-8F cells in Colony formation assay and BrdU cell proliferation assay. **(A)** The images and the statistics exhibited that the number of clone cells in the L.v-shEIF3C group was less than that in the L.v-shCtrl group. **(B)** The magnitude of the absorbance for the developed color in the L.v-shCtrl group was greater than that in the L.v-shEIF3C group detected at 450 nm. The data is the means ± SDs of three independent experiments. The differences were statistically significant. **p < 0.01.

Cellomics was used to analyze the cell growth by taking pictures of cells with green fluorescent protein (GFP) came from L.v-shEIF3C and L.v-shCtrl. Images taken for five consecutive days, as well as the number of cells with fluorescence statistics exhibited that the number of cells infected with L.v-shEIF3C was remarkably less than another group (Figure 2). In the colony formation assay (Figure 3A), the number of clone cells in the two groups was counted, and it was found that the number of clone cells in the L.v-shCtrl group was higher than that in the L.v-shEIF3C group. Likewise, in the BrdU cell proliferation assay (Figure 3B), we found that the magnitude of the absorbance for the developed color in the L.v-shCtrl group was greater than that in the L.v-shEIF3C group detected at 450 nm. These results suggested that down-regulation of EIF3C can inhibit cell proliferation and retard tumor growth.

### Down-regulation of EIF3C promoted the apoptosis of FaDu and 5-8F cells by regulating the protein expression of key genes in the signaling pathways

**FIGURE 4:**
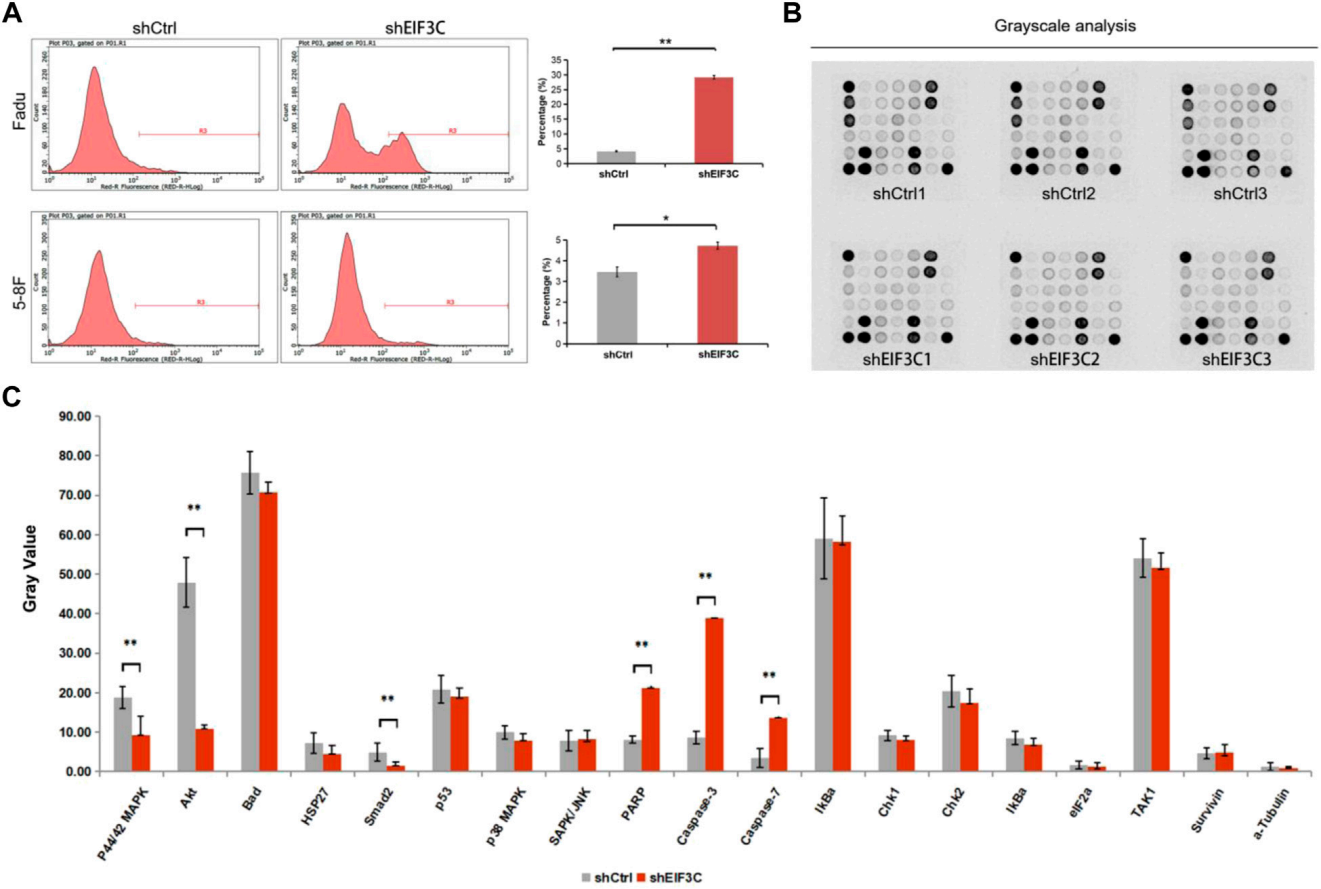
The L.v-shEIF3C promoted the apoptosis of FaDu and 5-8F cells. **(A)** The flow cytometry analysis showed that the apoptotic cells in the L.v-shEIF3C group were more than that in the L.v-shCtrl group. **(B)** Images were captured by exposing the slide slightly to standard chemiluminescent film. **(C)** Pathscan sandwich ELISA was used to detect the protein expression of key genes in apoptosis-related signaling pathways, and the results showed that the expression of phospho-P44/P42 MAPK, phospho-Akt and phospho-Smad2 was decreased, the expression of cleaved-PARP, cleaved-Caspase3 and cleaved-Caspase7 was increased by L.v-shEIF3C. The data is the means ± SDs of three independent experiments. The differences were statistically significant. *p < 0.05, **p < 0.01.

Flow cytometry was used to analyze the apoptosis when FaDu and 5-8F cells were infected with L.v-shEIF3C and L.v-shCtrl, it was found that the number of apoptotic cells in L.v-shEIF3C group was significantly more than that in L.v-shCtrl group (Figure 4A), indicating that down-regulation of EIF3C can promote apoptosis of tumor cells. In order to further study the regulation mechanism of EIF3C on apoptosis, we used PathScan^®^ Stress and Apoptosis Signaling Antibody Array Kit to detect the protein expression of key genes in apoptosis-related signaling pathways, and found that the expression of six key genes was affected after the down-regulation of EIF3C (Figures 4B,C). The expression of phospho-P44/P42 MAPK, phospho-Akt, and phospho-Smad2 was decreased, the expression of cleaved-PARP, cleaved-Caspase3 and cleaved-Caspase7 was increased, which provided strong evidence that EIF3C regulated cell apoptosis.

### Downregulation of EIF3C triggered cell cycle arrest in FaDu and 5-8F cells

**FIGURE 5:**
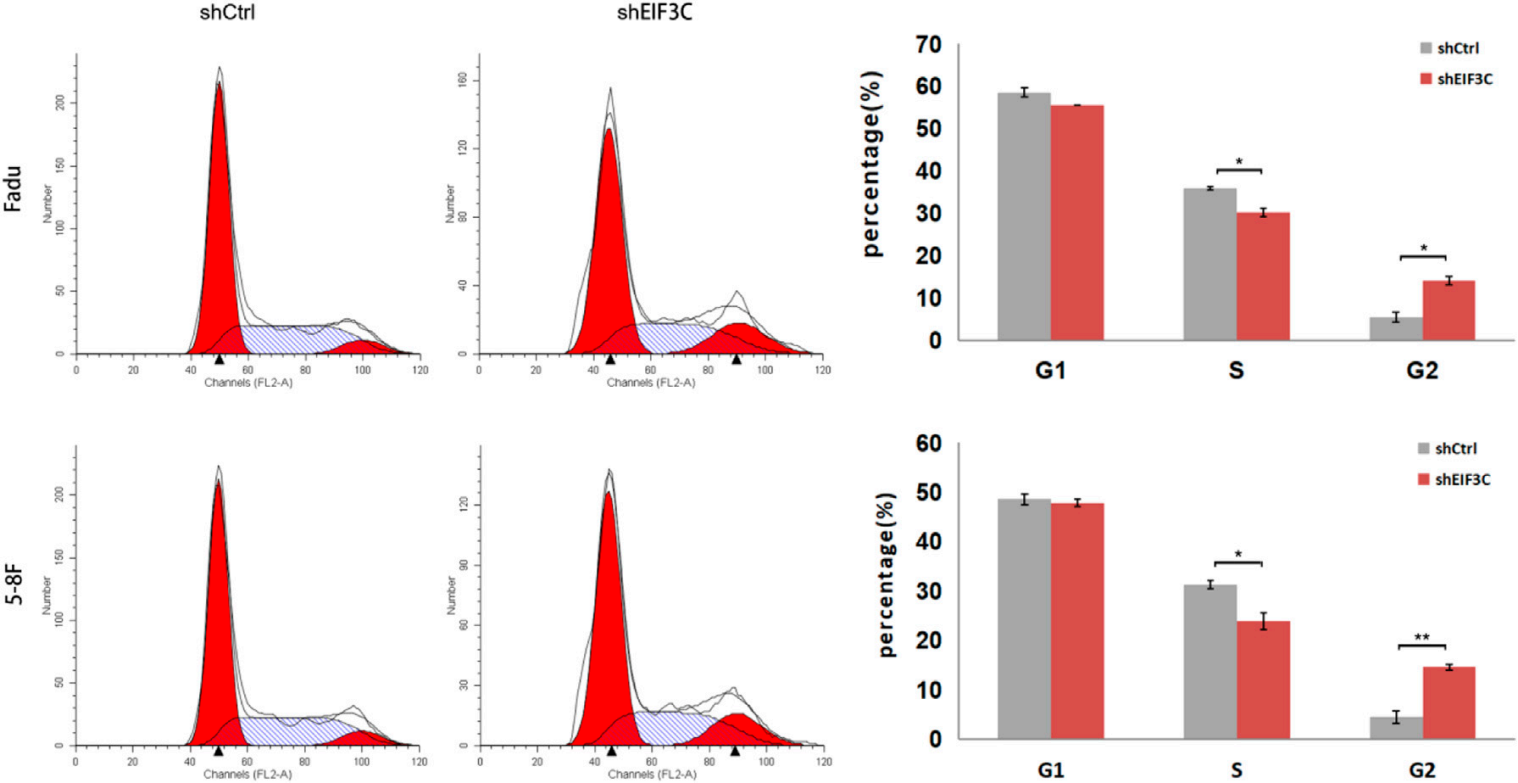
The L.v-shEIF3C triggered cell cycle arrest in FaDu and 5-8F cells. The flow cytometry analysis showed that the number of cells increased in G2 phase and decreased in S phase in L.v-shEIF3C group compared with the L.v-shCtrl group. The data is the means ± SDs of three independent experiments. The differences were statistically significant. *p < 0.05.

The cell cycle of FaDu and 5-8F cells infected with L.v-shEIF3C and L.v-shCtrl were analyzed by Flow cytometry, the results showed that the number of cells in the G2 phase increased and the number of cells in the S phase decreased in L.v-shEIF3C group compared with the L.v-shCtrl group (Figure 5), suggesting that EIF3C was significantly correlated with the cell cycle distribution of FaDu and 5-8F cells, and down-regulation of EIF3C could trigger the cell cycle arrest of tumor cells.

### L.v-shEIF3C suppressed tumor formation and growth in a FaDu cancer cell xenograft animal model

**FIGURE 6:**
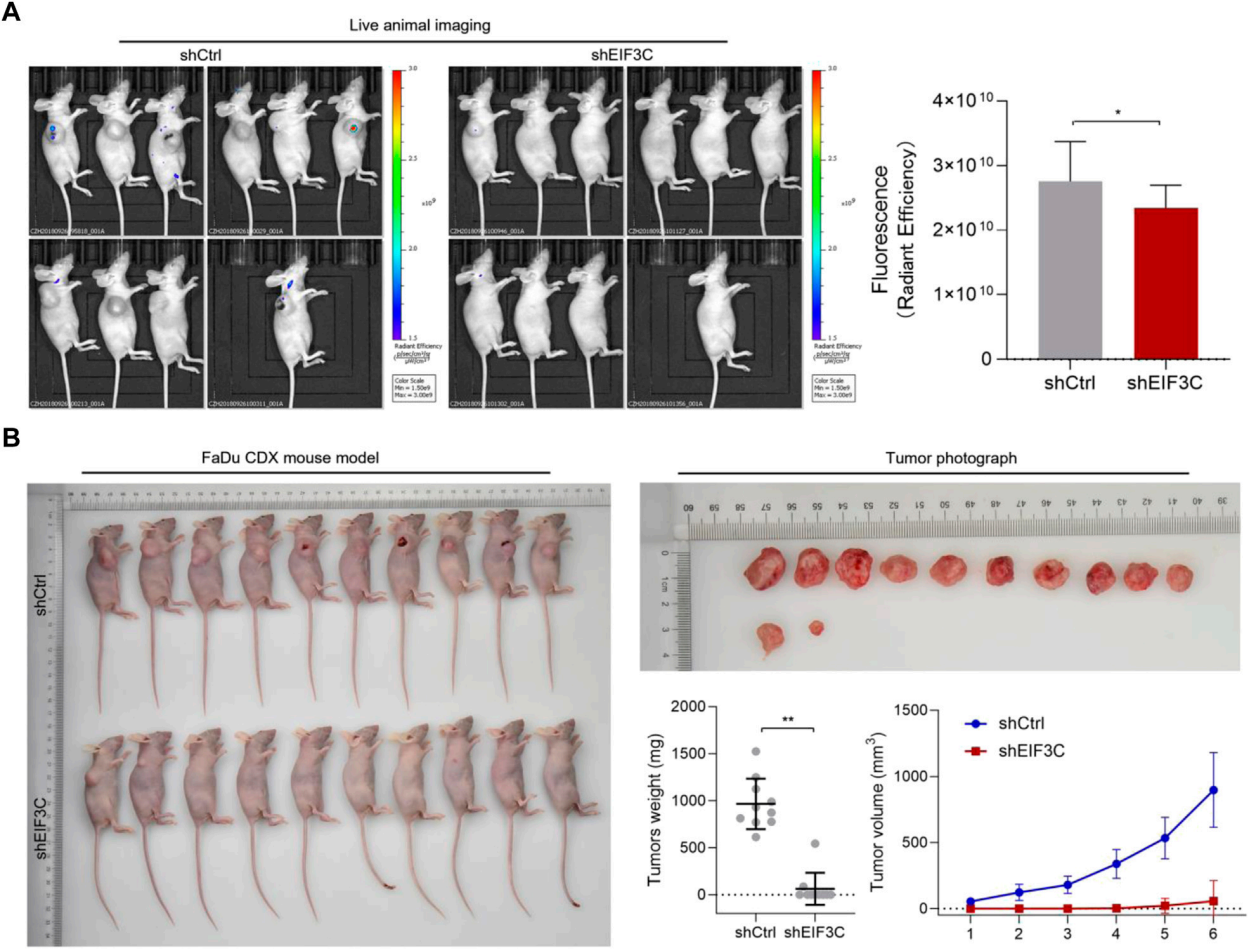
L.v-shEIF3C suppressed tumor formation in a FaDu cancer cell xenograft animal model. **(A)** live animal imaging images and the analysis showed that the fluorescence intensity of tumor in the L.v-shCtrl group was higher than that in L.v-shEIF3C group. **(B)** The photos of mice and tumors, as well as tumor weight and size statistics showed that the tumors were small in size and low in weight in L.v-shEIF3C group compared with the L.v-shCtrl group. The data is the means ± SDs. The differences were statistically significant. *p < 0.05, **p < 0.01.

A FaDu mouse xenograft model was constructed to study the effect of EIF3C on tumor *in vivo*, FaDu cells were inoculated into the back of mice, tumor size was measured and recorded with vernier calipers every 3 days from the 14th day after inoculation until the 32nd day. On the last day, the tumor growth in mice was observed by small animal imager, and the fluorescence intensity of tumor was statistically analyzed. It was found that the fluorescence intensity of tumor in the control group was significantly higher than that in the experimental group (Figure 6A). Then the mice were sacrificed, the tumor masses were taken out and photographed, and the tumor masses were weighed. Through photo observation and volume and weight analysis of the tumor masses (Figure 6B), it was found that the tumor masses in the experimental group were small in size and low in weight, indicating that down-regulation of EIF3C could inhibit the formation and growth of tumor cells in mice.

## Discussion

In this study, the role of EIF3C in head and neck cancer was investigated by constructing shRNA to down-regulate EIF3C expression. The results showed that down-regulating EIF3C inhibited the proliferation of FaDu and 5-8F cells, promoted their apoptosis, induced cycle arrest, and inhibited tumor growth in a mouse xenograft model. It has been shown that reduced EIF3C expression in melanoma and ovarian adenocarcinoma can mediate G0/G1 or G2/M phase arrest and lead to reduced cell proliferation and ultimately cell death in a tissue-dependent manner (Emmanuel et al., 2013), which was consistent with our findings in head and neck cancer, and thus, our study also provided another important evidence for the role of EIF3C in tumor development.

Recent studies have shown that the main reason of poor outcomes of patients with advanced head and neck cancer is the uncontrolled migration and invasion of cancer cells, the metastases of cancer cells make it difficult to eradicate with a single surgical treatment or regional chemoradiotherapy (Bossola, 2015; Kaidar-Person et al., 2018). At present, it has become a hot research direction to search for tumor differential genes and diagnostic markers. It is significant to study the biological process precisely regulated by cytokines in tumor formation and development and the regulation of gene translation for the diagnosis and treatment of tumors (Konings et al., 2020; Kordbacheh and Farah, 2021).

EIF3C, one of the core subunits of the eukaryotic translation initiation factor EIF3 complex, is critical in the process of translation initiation, and studies have shown that down-regulation of EIF3C affects overall cellular protein synthesis, thereby interfering with cellular function. Therefore, we examined the protein expression levels of several genes in the apoptosis-related signaling pathway through pathscan sandwich ELISA in this study, and found that the expressions of phosphorylated P44/p42MAPK, phosphorylated AKT and phosphorylated Smad2 were down-regulated. P44 and P42 MAP kinase (ERK1 and ERK2) play important roles in regulating cell growth and differentiation (Plotnikov et al., 2011; Sabio and Davis, 2014). The serine/threonine kinase AKT is a proto-oncogene and a major medical concern that plays an important role in regulating multiple different cellular functions, including metabolism, growth, proliferation, survival, transcription, and protein synthesis (Porta et al., 2014; Ersahin et al., 2015). The protein encoded by Smad2 gene belongs to the SMAD protein family, which is a regulator of signal transduction and transcription in a variety of signaling pathways, and the proteins can regulate a variety of cellular processes, such as cell proliferation, apoptosis and differentiation by mediating transforming growth factor (TGF-β) signaling (Yang et al., 2019). Therefore, down-regulating EIF3C affected the expression of phosphorylated P44/p42 MAPK, phosphorylated AKT and phosphorylated SMad2, which promoted the FaDu cells apoptosis by down-regulating the protein expression of these three genes. In contrast, when cell apoptosis occurs, PARP, an inhibitor of DNA damage repair enzymes, was cleaved, and caspase-3 and caspase-7 were activated, the protein expression of cleaved-PARP, cleaved-caspase-3 and cleaved-caspase-7 was up-regulated, all indicating the onset of apoptosis (Shi, 2002; Cohausz and Althaus, 2009; Zhang et al., 2015; Xu et al., 2019).

In summary, we investigated the role of EIF3C in pharyngeal squamous carcinoma and nasopharyngeal carcinoma cells, and combined with animal models, we found that down-regulation of EIF3C could inhibit tumor progression in head and neck cancer, which provides a basis for evaluating EIF3C as a potential diagnostic or prognostic marker of head and neck cancer. The pathscan sandwich ELISA also preliminarily explored the protein expression signaling pathway associated with EIF3C affecting the apoptosis in pharyngeal squamous carcinoma cells, however, the specific regulatory mechanism of EIF3C in head and neck cancer remains to be further investigated.

## Data Availability

The original contributions presented in the study are included in the article/supplementary material, further inquiries can be directed to the corresponding author.
